# Annular pigment band on the posterior capsule following blunt ocular trauma: a case report

**DOI:** 10.1186/1471-2415-5-13

**Published:** 2005-06-20

**Authors:** Aashish Anand, Rosalind J Harrison

**Affiliations:** 1 Department of Ophthalmology, Queen's Hospital NHS Trust, Belvedere Road, Burton-on-Trent, DE130RB, UK

## Abstract

**Background:**

To report an unusual case of annular pigment band on the posterior capsule following blunt ocular trauma.

**Case presentation:**

We describe an annular pigment band on the posterior capsule following blunt ocular trauma in a 28-year old male patient. Repeat examinations revealed no evidence of other signs of blunt ocular trauma or pigment dispersion syndrome in either eye.

**Conclusion:**

The annular pigment band in this case corresponds to the adherence of the hyaloideocapsulare ligament to the posterior capsule and reconfirms its rare visualization in the living eye. This finding may be an isolated sign of blunt ocular trauma and a compromised integrity of the vitreolenticular interface should be strongly suspected. We recommend careful documentation in context of future cataract surgery in these eyes.

## Background

A blunt ocular trauma results in antero-posterior compression of the globe with simultaneous expansion in the equatorial plane. The impact is primarily absorbed by the lens-iris diaphragm and the vitreous base. Several changes have been described in the crystalline lens following blunt trauma. Direct and indirect forces may lead to contusion cataracts, lens subluxation/dislocation and Vossius'ring pigment deposition.

There are reports of isolated posterior capsule rupture [1,2], detachment of posterior capsule [3] and intralenticular hemorrhage [4] following blunt trauma. We report the first case of an isolated annular pigment band on the posterior capsule following blunt ocular trauma in a young male.

## Case presentation

A 28-year-old male presented with a dull ache and photophobia in the right eye following blunt ocular trauma. Three days earlier he had been hit in the right eye with a cricket ball. There were no other ocular complaints or significant past ocular history. The patient was in excellent general health and was not taking any medications.

On examination, his corrected visual acuities were 6/12 and 6/6 in the right and left eye respectively. Intraocular pressures were 19 mm Hg in each eye. Biomicroscopy revealed a moderate traumatic iritis in the right eye. After pupillary dilatation, ocular examination revealed a clear lens with an annular pigment band on the posterior capsule in the right eye. No other signs of blunt trauma were detected on a detailed examination of the anterior and posterior segments. The examination of left eye was within normal limits. There was no evidence of any pigmentation on the posterior capsule in the left eye. The patient was treated with mild steroids (prednisolone acetate 0.5% q.i.d) and cycloplegics (cyclopentolate 1%) for a week.

During follow-up evaluation of the patient after two weeks, visual acuities were 6/6 in both eyes. The traumatic iritis in the right eye had resolved. On a detailed evaluation of the right eye, no sequelae of blunt trauma were found. However, the annular pigment band on the posterior capsule of right eye persisted. A directed examination was performed in both eyes for signs of pigment dispersion syndrome. A re-examination of posterior corneal surface did not reveal Krukenberg's spindle or any other pigment deposition patterns in either eye. In both eyes, the anterior chamber was of normal depth with no noticeable concavity of the peripheral iris. There were no transillumation defects in the iris on careful retroillumination. Gonioscopy revealed open angles without any evidence of hyperpigmentation or angle recession in either eye. The cup to disc ratios were 0.3 in both eyes with a healthy neuroretinal rim. On direct questioning, there was no significant family history of glaucoma or other ocular disorders.

## Discussion

To the best of our knowledge and based on a MEDLINE search, such a presentation following blunt ocular trauma has not been reported.

Hyaloideocapsulare ligament of Weiger is an annular attachment of the anterior cortical vitreous to the posterior lens capsule. It is strongest in the midperipheral region of the lens. Although the existence of this circular adherence is widely described in the textbooks of ocular anatomy, Weiger's ligament is normally invisible in the living eye. A prominent, circular retrolental line corresponding to the Weiger's ligament has been rarely observed biomicroscopically in the living eye [5]. We propose that the annular pigment band on the posterior capsule in our patient was the result of posttraumatic pigment scattering and deposition in the crevice (sinus hyaloideocapsulare), formed just anterior to the Weiger's ligament. The annular shape of pigment band could correspond to the adherence of the Weiger's ligament to the posterior capsule. This observation reconfirms the rare visualization of Weiger's ligament in the living eye.

Pigmentation of posterior lens capsule, classically in the shape of a line (Scheie's line or Zentmayer's line) has been reported to be associated with pigment dispersion syndrome [6-8]. We believe that pigment dispersion syndrome can be ruled out as the cause of pigment deposition in our patient because of absence of any other clinical signs of pigment dispersion in either eye on a directed examination. In addition, unilateral involvement is more consistent with an episode of traumatic insult.

An unexpected smooth oval defect in the posterior capsule has rarely been encountered intraoperatively during uneventful traumatic cataract removal in the young [1,2]. The outlines of these defects were consistent with the border of Weiger's ligament. It is equally plausible that the pigment band on the posterior capsule in our patient may be a subtle sign of blunt trauma forces having acted in the central region of posterior capsule and a compromised integrity of this vitreolenticular interface. We recommend a careful documentation of this observation following blunt ocular trauma since it may be relevant in the context of future cataract surgery in these eyes.

## Competing interests

The author(s) declare that they have no competing interests.

## Authors' contributions

AA and RJH reviewed the patient in the clinic. AA conceived the study and prepared the manuscript. All authors read and approved the final manuscript.

## Pre-publication history

The pre-publication history for this paper can be accessed here:



## Figures and Tables

**Figure 1:**
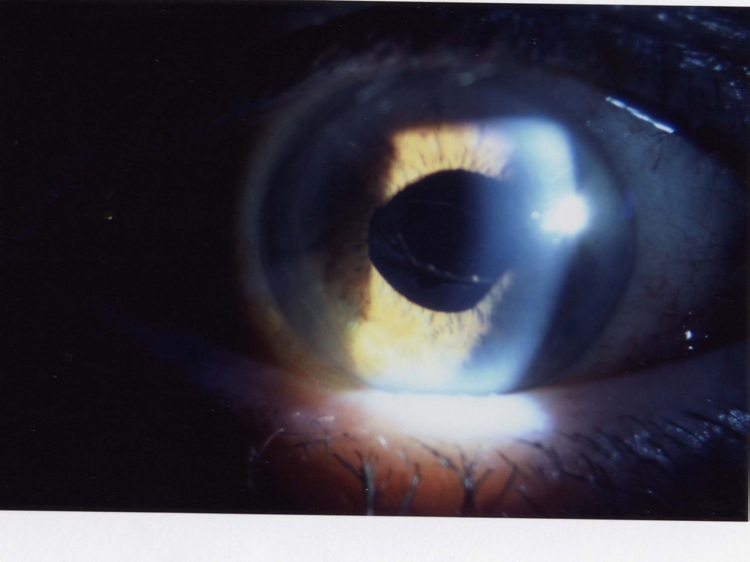
Annular pigment band on the posterior capsule.

**Figure 2:**
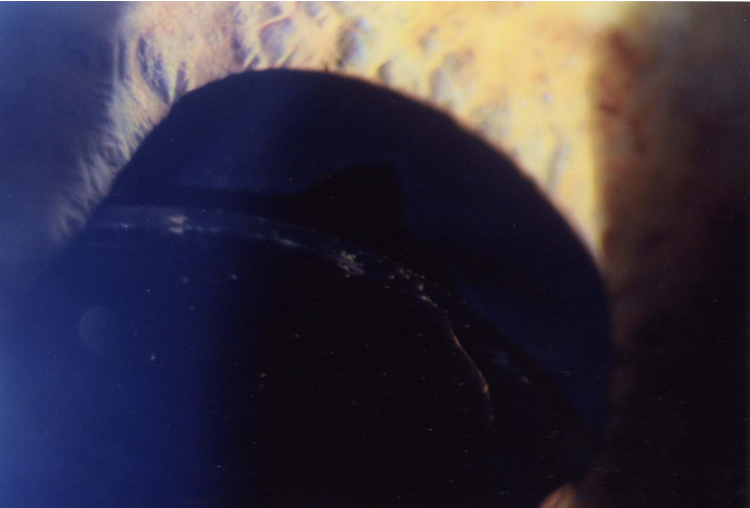
Annular pigment band on the posterior capsule: a magnified view.
